# Can community health workers increase modern contraceptive use among young married women? A cross-sectional study in rural Niger

**DOI:** 10.1186/s12978-019-0701-1

**Published:** 2019-03-25

**Authors:** Mohamad I. Brooks, Nicole E. Johns, Anne K. Quinn, Sabrina C. Boyce, Ibrahima A. Fatouma, Alhassane O. Oumarou, Aliou Sani, Jay G. Silverman

**Affiliations:** 1 Pathfinder International, Watertown, MA 02472 USA; 2 Center on Gender Equity and Health, University of California, San Diego, La Jolla, CA 92093 USA; 3 Pathfinder International, Niamey, Niger

## Abstract

**Background:**

The Republic of Niger has the highest rate of early marriage and adolescent fertility in the world. Recent global health initiatives, such as Family Planning 2020, have reinvigorated investments in family planning in low- and middle-income countries (LMICs). As part of this initiative, Niger has implemented ambitious plans to increase contraceptive prevalence through policies designed to increase coverage and access to family planning services. One strategy involves the deployment of volunteer community health workers (*relais communautaires*) in rural settings to improve access to family planning services, especially among adolescents and youth. The objective of this article is to determine if visits by *relais* are associated with increased use of modern contraception among young married women in rural Niger.

**Methods:**

Cross-sectional data from a household survey were collected from young married women between the ages of 13 and 19 in three rural districts in the region of Dosso, Niger from May to August 2016. Multivariate logistic regression was conducted to assess the odds of married female youth reporting current use of modern contraceptive methods based on being visited by a *relais* in the past three months.

**Results:**

A total of 956 young married women were included in the final analysis. Among study participants, 9.3% reported a *relais* visit to discuss health issues in the past three months and 11.4% reported currently using a modern method of contraception. Controlling for socio-demographic variables, the odds of current use of modern contraceptive methods were higher among young married women who were visited by a *relais* in the last three months compared to those not visited by a *relais* during this period (AOR = 1.94[95% CI 1.07–3.51]). In this study setting, *relais* were less likely to visit nulliparous women and women that worked in the past 12 months.

**Conclusion:**

Young married women visited by *relais* were more likely to use modern contraceptive methods compared to those not visited by a *relais*. These results are consistent with similar family planning studies from sub-Saharan Africa and suggest that *relais* in Niger may be able to provide access to essential family planning services in rural and hard-to-reach areas. Additional efforts to understand the contraceptive barriers faced by nulliparous women and working women should be a key research priority in Niger.

**Trial registration:**

Clinical trial registration number 2016–1430; registered on October 7, 2016 (retrospectively registered).

## Abstract (fr)

**Contexte:**

La République du Niger a le taux le plus élevé de mariages précoces et de fécondité des adolescentes du monde. Les récentes initiatives mondiales pour la santé, telles que Planification Familiale 2020 (FP2020), ont redynamisé les investissements dans la planification familiale dans les pays à revenu faible ou intermédiaire. Dans le cadre de cette initiative, le Niger a mis en œuvre des plans ambitieux pour accroître la prévalence de la contraception par le biais de politiques conçues pour accroître la couverture et l’accès aux services de planification familiale. Une des stratégies consiste au déploiement d’agents de santé communautaires volontaires (*relais communautaires*) en milieu rural pour améliorer l’accès aux services de planification familiale, en particulier chez les adolescents et les jeunes. L’objectif de cet article est. de déterminer si les visites à domicile conduites par des *relais* sont associées à une utilisation accrue de la contraception moderne chez les jeunes femmes mariées des zones rurales du Niger.

**Méthodes:**

Les données transversales d’une enquête-ménages ont été collectées auprès de jeunes femmes mariées âgées de 13 à 19 ans dans trois districts ruraux de la région de Dosso, au Niger, de Mai à Août 2016. Une régression logistique multivariée a été menée pour évaluer la probabilité que les jeunes femmes mariées déclarant utiliser actuellement des méthodes contraceptives modernes après avoir été visitées par un relai au cours des trois derniers mois.

**Résultats:**

Au total, 956 jeunes femmes mariées ont été incluses dans l’analyse finale. Parmi les participants à l’étude, 9,3% ont signalé une visite de relais pour discuter de problèmes de santé au cours des trois derniers mois et 11,4% ont déclaré utiliser actuellement une méthode de contraception moderne. En tenant compte des variables sociodémographiques, les probabilités d’utilisation actuelle de méthodes contraceptives modernes étaient plus élevées chez les jeunes femmes mariées visitées par un relai au cours des trois derniers mois par rapport à celles qui n’avaient pas été visitées par un relai au cours de cette période (AOR = 1,94 [95% IC 1,07-3,51]). Dans cette étude, les relais étaient moins susceptibles de rendre visite aux femmes nullipares et aux femmes ayant travaillé au cours des 12 derniers mois.

**Conclusion:**

Les jeunes femmes mariées visitées par les relais étaient plus susceptibles d’utiliser des méthodes de contraception modernes que celles qui n’étaient pas visitées par les relais. Ces résultats concordent avec ceux d’études similaires sur la planification familiale menées en Afrique subsaharienne et suggèrent que les relais au Niger pourraient donner accès à des services essentiels de planification familiale dans les zones rurales et difficiles d’accès. Des efforts supplémentaires pour comprendre les barrières contraceptives rencontrées par les femmes nullipares et les travailleuses devraient constituer une priorité de recherche essentielle au Niger.

## Plain English summary

The West African nation of Niger has low family planning use, high rates of early marriage, and most of its population lives in rural areas—where there are fewer easily accessible health resources. In such countries with low investments in health, the deployment of volunteer community health workers (*relais communautaires*) is a common strategy used to improve access to family planning services, especially in rural settings. To our knowledge, no studies have explored patterns between *relais* visits and use of family planning in Niger. The study aimed to investigate if visits by *relais* are linked with increased use of modern family planning methods among young married women under the age of 20 years in Niger’s rural Dosso region. Results show that use of modern family planning methods almost doubled among young married women that were visited by a *relais* compared to those not visited by a *relais*. Results also show that *relais* were less likely to visit women with no children. Women who worked outside of their house in the last 12 months were also found to be less likely to use family planning. These findings suggest that *relais* contribute positively to access to and uptake of essential family planning services in Niger’s Dosso region but that further investigation is required to understand the challenges faced by young women without children and those that work when seeking family planning services.

## Background

In 2014, approximately 225 million women of reproductive age in developing countries had an unmet need for modern contraception, many of them living in rural and hard-to-reach areas with limited access to high-quality health services [1–3]. The use of modern contraception helps women avoid unintended pregnancies, unsafe abortions, and improves overall maternal, newborn, and child health [4, 5]. The benefits of contraception and family planning services go beyond health gains, including contributing to other Sustainable Development Goals (SDGs) such as reducing poverty (SDG 1), hunger (SDG 2), and inequalities (SDG 10), and improving education (SDG 4), gender equality (SDG 5), economic growth (SDG 8), and sustainable communities (SDG 11) [6, 7]. Investment in family planning is a “best buy” for global development efforts—without access to family planning services, including modern contraception, the impact and effectiveness of other interventions will be less, cost more, and will take longer to achieve [6, 8]. A recent post-2015 development agenda report written by the Copenhagen Consensus Center stated that a return of $120 USD would be achieved for every dollar spent on achieving universal access to sexual and reproductive health services by 2030 and eliminating unmet need for modern contraception by 2040 [9]. As result, recent initiatives have reinvigorated investments in family planning. For example, the Family Planning 2020 global partnership launched in 2012 aims to add 120 million new users of modern contraceptives in the world’s 69 poorest countries by 2020 [10]. While there have been important successes, such as a global increase in contraceptive use among married women, family planning investments and access still fall short in many low- and middle-income countries (LMICs).

The Republic of Niger is a low-income country in western sub-Saharan Africa. In 2014, the country spent 5.8% of its GDP on total health expenditure (THE), and had a THE per capita of US$54, one of the lowest in the world [11]. Although primary school completion rate is 69%, only 19% of adults are literate [11]. Out of a total of 189 countries listed, Niger ranked last for both the Human Development Index and Gender Inequality Index [12] indicating that major health and societal challenges, including maternal, neonatal, child, and adolescent health issues, persist. As part of the Family Planning 2020 initiative, the Government of Niger has committed to raise its contraceptive prevalence rate from 12% in 2012 to 50% in 2020 [10].

Adolescent girls in Niger have the highest adolescent fertility rate in the world, which is driven in part by the high rates of early marriage—by the age of 18 years, 59% of girls have already been married [13]. Immediately after marriage, girls face pressure to bear children, resulting in one of the highest rates of early childbearing in the world. Among women between the ages of 20 and 24 years in Niger, 9% gave birth before the age of 15 and 51% gave birth before the age of 18 [13]. After the first birth, social norms supportive of frequent childbearing and gender norms that restrict a woman’s ability to make choices about her health and fertility encourage adolescents to have closely spaced pregnancies, consequently increasing risk for their health and the health of their infants. Furthermore, modern contraceptives are not widely used; only 5.9% of married adolescent girls in Niger between the ages of 15 and 19 years use a modern method [14]. These factors contribute to Niger’s total fertility rate of 7.6 children per woman—the highest in the world [14].

The problem of low access and utilization of modern contraceptive experienced by adolescent girls in Niger can be resolved to some degree by the introduction of Community Health Workers (CHW). In settings where unmet need for family planning is high and access is low, evidence has shown that CHWs can increase uptake and use of contraception [15]. In Niger, CHWs are health providers who lack formal medical training but have been trained to provide “health education, referral and follow-up, case management, basic preventive health care, and home visiting services” in the communities in which they live [16]. In a recent systematic review to evaluate the strength of evidence for the provision of family planning services by CHWs, 50 out of 54 studies with robust study designs—such as randomized control trials, longitudinal studies with comparison groups, and pre- and post-test studies—showed that CHWs effectively increased contraceptive use [17]. In a sub-analysis of studies that compared rates of current use of modern contraceptives between CHW intervention groups and non-CHW intervention groups, CHW intervention groups in these studies experienced a two-fold higher average increase in modern contraceptive use from baseline to follow-up compared to the non-CHW groups [17].

Niger has an active cadre of volunteer CHWs called *relais communautaires*. *Relais* are volunteers chosen by their communities to perform a number of tasks related to health promotion, treatment, and linkages with formal health care facilities [18]. *Relais* are selected based on their ability to read and write, their dynamic and social personality, and must be a member of that community over the age of 20. They are trained by health personnel, primarily from local and international non-governmental organizations, in a range of health, family planning, nutrition, hygiene, and sanitation issues. In addition, *relais* are provided with basic pharmaceutical products, family planning commodities, and other first aid equipment that they can then distribute to members of their communities. *Relais* work under the supervision of the head of the local health unit and in close coordination with non-governmental organizations [18].

There is a dearth of empirical evidence that study and document the effects and associations of family planning programs and interventions in Niger. To our knowledge, no studies have explored patterns between *relais* visits and use of contraception in Niger. This manuscript will contribute to the limited evidence base of family planning studies that currently exist in Niger. In a country with low investments in health, high rate of adolescent fertility, low contraceptive use, and restrictive social and gender norms, exploring *relais’* effect on contraceptive use among adolescent girls is an important public health topic both in Niger and other West African countries. The objective of this analysis is to determine if visits by *relais* are associated with increased use of modern contraception among young married women in rural Niger.

## Methodology

### Data collection

A cross-sectional household survey was implemented among young married women ages 13 to 19 years and their husbands in the region of Dosso, located in the south western corner of Niger, from May to August 2016. Ethical review and approval of this study was obtained from the University of California, San Diego and the Republic of Niger Ministry of Health institutional review boards (IRBs). Informed consent to participate in the study was obtained for all participants recruited for this study.

A two-stage cluster sampling approach was used in this study. Data were collected across 48 villages clustered within the Dosso, Doutchi, and Loga districts in Dosso region. Villages were randomly selected among all of those meeting the following inclusion criteria: 1) having at least 1000 permanent inhabitants; 2) primarily Hausa or Zarma-speaking (the two major languages of Niger); and 3) including no known recent intervention specifically around family planning (beyond standard health services and including CHW visits) or female empowerment with adolescent wives or their husbands. Based on this village inclusion criteria, there was a total of 104 eligible villages in Dosso district, 114 eligible villages in Doutchi district, and 32 eligible villages in Loga district. In the first stage, quota random sampling was performed so that 3 villages with health facilities, 9 villages with health posts, and 4 villages with neither a health facility or health post, were randomly selected from each district for a total of 48 villages (16 villages in each district). In the second stage, 25 young married women from each of the 48 villages (*N* = 1200) were randomly selected using simple random sampling from a list of all married women living in the village that was provided by the village chief. Eligibility criteria for participants include: 1) adolescent women ages 13 to 19 years old; 2) married; 3) fluent in Hausa or Zarma; 4) residing in the village where recruitment was taking place with no plans to move away in the next 18 months or plan to travel for more than 6 months during that period; 5) not currently sterilized; and 6) providing informed consent to participate in the study. Trained enumerators conducted the interviews with study participants in a private setting and read the questions aloud in either Hausa or Zarma. Of those who were randomly selected, 88.0% of young married women participated in the household survey for a final sample size of 1097 participants. Randomly selected young married women from the village chief’s list were used as replacements when the originally selected woman was unavailable or ineligible for participation. No significant differences in wife age, husband age, or time spent away from the village were observed across those who did and did not participate. This analysis excluded women who were pregnant or did not know their pregnancy status (*n* = 140) and women missing all outcome data (n = 1) for a final analytic sample of 956 participants.

### Key variables

#### Dependent variables: current family planning use

The dichotomous outcome variable representing current use of modern family planning methods was constructed based on the type of family planning women reported to be currently using in the past three months. Modern family planning use was coded “yes” if participants reported any of the following types of contraception: intrauterine device (IUD), injectables, implants, oral contraceptive pill, condom, female condom, emergency contraception (EC), or lactation amenorrhea method (LAM). LAM was categorized as a modern family planning method in order to align with the modern contraceptive definitions provided by the international Demographic Health Surveys and National Institute of Statistics in Niger [14]. If a woman reported using any traditional methods, abstinence, or was not currently using any type of family planning method, modern family planning use was coded as “no”.

#### Independent variable: Relais visit(s) in past three months

*Relais* visit(s) in the past three months was dichotomously measured by asking women if any *relais* visited their home and talked to them about health issues in the past three months.

#### Sociodemographic covariates

Sociodemographic data was collected as part of the household survey. The wife’s age in years was categorized as “very young” (under 16 years), “younger adolescent” (16 to 17 years), and “older adolescent” (18 to 19 years). Husband’s age in years was coded as a binary variable, “youth” (15 to 24 years) and “adult” (25 years and older).

Parity was asked directly of wives; where missing, number of living children born to the wife was used as a proxy for parity.

Education type of wives and husbands was grouped into three mutually exclusive categories: “government school,” “Quranic school,” and “no school.” Participants who reported both modern and Quranic school attendance were classified as affirmative for “government school;” “Quranic school” contains only the respondents who attended a Quranic school but did not attend a government school.

Family wealth was assessed using the standard household asset list from the Niger 2012 Demographic and Health Survey [14], which includes the presence of the following items in the participants’ home: watch, mobile phone, bicycle, motorbike or scooter, car or truck, or an animal drawn cart. A composite variable of the summation of these assets was created; this composite score was then dichotomized as “less than median household assets score” and “median and above household asset score.”

Experiencing food insecurity was coded as affirmative if the respondent reported in the last month that anyone in the household went without eating the whole day because there was not enough food. Additional sociodemographic covariates included whether the husband spent most nights at the compound where the woman lived, the husband’s number of wives, whether the woman had engaged in work outside of the home in the past 12 months, whether the husband was away from their village for more than 3 months in the past year, tribe affiliation of the couple, and the district where the woman currently resides.

### Data analysis

Multivariate logistic regression was conducted using data from young married women who were not pregnant at the time of the survey to assess whether reporting current use of modern family planning methods was significantly associated with being visited by a *relais* in the past three months. Bivariate chi-squared tests were run to assess the individual associations of the sociodemographic covariates with current use of modern family planning methods. All covariates with a chi-squared *p*-value of < 0.20 were included in an adjusted model. We used a p-value cut-off point of < 0.20 as more traditional levels such as < 0.05 can fail in identifying variables known to be important [19]. Because of collinearity between tribe and district, tribe was omitted despite association at *p* < 0.20. The final parsimonious model was constructed from this adjusted model using backwards selection at a threshold of *p* < 0.10.

## Results

A total of 956 young married women were included in the current analyses (Table 1). Slightly over half (53.3%) of women in this study population were older adolescents (18 to 19 years old) and about half (48.9%) had no formal education. In regards to the husbands of the surveyed women, about half (48.1%) were under the age of 25, about two-thirds (68.4%) had either modern or Quranic schooling, and approximately 1 in 7 (13.3%) had more than one wife. In addition, the majority (69.8%) of husbands spent a period longer than three months away from the village. In this rural setting, about one third (32.9%) of young married women lived in households with less than the median household asset score and about a quarter (22.5%) experienced food insecurity in the past month.

**Table 1:** Characteristics of *relais* visits in the past three months and current use of modern contraceptive methods among young married women in Dosso region, Niger 2016

Sociodemographic covariates^a^	Overall		Visited by *relais* in past three months		Current use of modern contraceptive methods	
	*N*	(%)	*n*	(%)	*n*	(%)
	956	(100)	89	(9.3)	109	(11.4)
Age						
13 to 15 (very young)	162	(16.9)	8	(9.0)	10	(9.2)
16 and 17 (younger adolescent)	284	(29.7)	19	(21.4)	22	(20.2)
18 and 19 (older adolescent)	510	(53.4)	62	(69.7)	77	(70.6)
Husband’s age						
Under 25	445	(48.1)	25	(29.8)	39	(36.4)
25 and older	481	(51.9)	59	(70.2)	68	(63.6)
Husband’s number of wives						
1	803	(86.7)	74	(88.1)	94	(87.9)
2	111	(12.0)	8	(9.5)	12	(11.2)
3 or more	12	(1.3)	2	(2.4)	1	(0.9)
Education level						
No school	464	(49.0)	33	(37.5)	47	(43.9)
Quranic school	166	(17.5)	19	(21.6)	26	(24.3)
Government school	318	(33.5)	36	(40.9)	34	(31.8)
Husband’s education level						
No school	291	(31.6)	23	(27.7)	26	(24.3)
Quranic school	193	(20.9)	19	(22.9)	33	(30.8)
Government school	438	(47.5)	41	(49.4)	48	(44.9)
Parity						
None	360	(37.7)	9	(10.1)	4	(3.7)
1 child	321	(33.6)	29	(32.6)	44	(40.4)
2 children	206	(21.5)	37	(41.6)	46	(42.2)
3 or more children	69	(7.2)	14	(15.7)	15	(13.7)
Worked in last 12 months						
Yes	407	(42.8)	30	(33.7)	39	(35.8)
Husband sleeps most nights in the same compound						
Yes	856	(90.0)	82	(92.1)	102	(93.6)
Husband spent a period longer than three months away from village						
Yes	644	(69.8)	49	(58.3)	76	(71.0)
Household Assets						
Less than median household asset score	304	(32.9)	34	(40.5)	36	(33.6)
Median or above household asset score	619	(67.1)	50	(59.5)	71	(66.4)
Food insecurity						
Yes	214	(22.5)	25	(28.1)	30	(27.5)
Tribe						
Zarma	650	(68.9)	51	(58.6)	59	(55.1)
Hausa	294	(31.1)	36	(41.4)	48	(44.9)
District						
Dosso	310	(32.4)	37	(41.6)	21	(19.3)
Doutchi	316	(33.1)	35	(39.3)	52	(47.7)
Loga	330	(34.5)	17	(19.1)	36	(33.0)

^a^ *Missing data not reported*

Approximately 1 in 10 (9.3%) young married women reported a *relais* visit in the past 3 months to discuss health issues. Among those visited by *relais*, the majority (69.7%) of visits were among older adolescent (18 to 19 years old). In addition, the great majority (89.9%) of *relais* visits took place among young married women with children.

Within this study population, 11.4% of young married women between the age of 13 and 19 years reported currently using a modern method of contraception. Current use of modern contraceptives was higher among older adolescents ages 18 to 19 years old (70.6% of the population using modern contraception) and those with children (96.3%). Among young married women visited by *relais* in the past three months, 24.7% (22/89) currently use modern contraceptives. In comparison, 10.0% (87/867) of young married women not visited by *relais* in the past three months currently use modern contraceptives.

A total of 89 young married women were visited by *relais* in the past three months (Table 2); over half (61.8%) had more than one *relais* visits. The majority of participants (89.9%) reported that the *relais* discussed family planning as part of their household visit, with approximately three-quarters (76.4%) of participants receiving family planning commodities as part of their visit. Oral contraceptives pills were the most common (68.7%) method provided. In the event young married women requested contraceptive commodities not provided by the *relais*, about half (51.7%) of participants reported that the *relais* accompanied them to a health clinic where they were provided with pills (53.3%) or a contraceptive injection (45.7%). The majority (94.4%) of young married women found the *relais* visits helpful, while only a small proportion (7.9%) stated that someone in their household was disapproving of the *relais* visit.

**Table 2:** Description of *relais* visits among young married women visited by *relais* in past three months in Dosso region, Niger 2016

	Overall	
	*N*	(%)
	89	(100)
Number of *relais* visits in past three months		
1	34	(38.2)
2	37	(41.6)
3	18	(20.2)
Topics discussed during *relais* visit		
Family Planning	80	(89.9)
Pregnancy Health	52	(58.4)
Nutrition	51	(57.3)
Gender	11	(12.4)
*Relais* provided family planning		
Yes	68	(76.4)
No	21	(23.6)
Type of family planning provided		
Pill	46	(68.7)
Condom	2	(3.0)
Female Condom	3	(4.5)
Other	23	(33.8)
*Relais* accompanied participant to health center		
Yes	46	(51.7)
No	43	(48.3)
Type of FP provided at health center		
Pill	24	(53.3)
Injectables	21	(45.7)
Implants	1	(2.2)
Other	3	(6.5)
Found *relais* visit helpful		
Yes	84	(94.4)
No	5	(5.6)
What *relais* information was helpful		
Helped me make choices about my life	67	(79.8)
Helped teach me how to healthily space my pregnancies	54	(64.3)
Helped me to speak to my husband about family planning	47	(56.0)
Helped to better understand how to keep me and my family healthy	17	(20.5)
Someone in household was disapproving of relais visit		
Yes	7	(7.9)
No	65	(73.0)
Don’t know	17	(19.1)

Results from the logistic regression analysis showed a positive association between *relais* visit in the last three months and current use of modern contraceptive methods (Table 3). Controlling for covariates, the odds of current use of modern contraceptive methods were higher in married adolescent youth who were visited by a *relais* in the last three months compared to those not visited by a *relais* during this period (AOR = 1.94 [1.07–3.51]). Increasing parity and residence in the Doutchi or Loga districts were also statistically significantly positively associated with current use of modern family planning in the regression model. Women who worked outside the home in the previous 12 months were significantly less likely to be currently using modern family planning.

**Table 3:** Adjusted odds ratios (AORs) and 95% confidence intervals (CIs) for predictors of current use of modern contraceptive methods among young married women in Dosso region, Niger 2016

	AORs†	95% CI	*P*-value
Independent variable			
Visited by *relais* in the last three months (ref = no)			
Yes	1.94	1.07,3.51	0.03*
Sociodemographic variables			
Parity (continuous)			
	1.94	1.59,2.37	< 0.001***
Husband’s education (ref = none)			
Quranic school	1.70	0.92,3.11	0.09
Government school	1.33	0.78,2.27	0.29
Worked in the last 12 months (ref = no)			
Yes	0.62	0.39,0.98	0.04*
District (ref = Dosso)			
Doutchi	2.76	1.55,4.92	0.001**
Loga	2.13	1.16,3.89	0.01*

*p < 0.05; **p < 0.01; ***p < 0.001

†Adjusted for all other covariates in the model. Covariates removed based on p-value of > 0.20 in bivariate chi-square tests include: husband’s number of wives, husband spent a period longer than 3 months away from village, household assets. Tribe was excluded because of collinearity with district. Covariates removed based on backwards model selection from full adjusted model with *p* > 0.10 include: wife’s age, husband’s age, wife’s schooling, husband spends majority of nights at wife’s compound, and food insecurity

## Discussion

The results of the study build upon earlier findings from sub-Saharan Africa that show positive association between CHW visits and current use of modern contraceptives [20–23]. The main findings show that *relais* visits are positively associated with current use of modern contraceptive methods among young married women in rural Niger (AOR = 1.94 [1.07–3.51]) which suggest that the deployment of *relais* may be a viable strategy in increasing utilization of modern contraception in this high-need context.

Results from the survey also showed that only 1 in 10 (10.1%) young married women who had a *relais* visit in the last 3 months were nulliparous. As a result, parity was positively associated with current use of modern family planning in the regression model (AOR = 1.94 [1.59–2.37]). These findings suggest that provider bias may be in effect in this setting of rural Niger. Other studies from sub-Saharan Africa have shown that provider bias is a key factor affecting uptake of contraceptive services among women, especially among adolescent, unmarried, or nulliparous women [24–26]. In particular, nulliparous women may face discrimination in receiving contraceptive counseling stemming from the cultural expectation that women need to prove their fertility early on in marriage [25, 26]. Additional efforts are needed to better understand the contraceptive barriers for nulliparous women in Niger and identify whether *relais* can provide family planning advice and contraceptive commodities to nulliparous women and younger adolescents in the communities that they support.

The regression model from this study showed a negative association between current use of modern family planning and employment (AOR = 0.62 [0.39–0.98]). This is a surprising finding as women’s empowerment, including proxy measurements such household decision making, mobility, education, and employment, are increasingly considered key factors affecting family planning and reproductive outcomes among women [27, 28]. In this setting, 42.8% of study participants stated that they worked outside the home in the last 12 months. It could be hypothesized that women who worked in this setting may be less likely to currently use modern contraceptive due to time constraints and conflicting priorities that limit access to family planning services. Additional research to better understand the dynamic between employment and contraceptive use among young married women in Niger is warranted.

Furthermore, the regression model also shows that young married women residing in Doutchi and Loga districts were positively associated with current use of modern family planning (AOR = 2.76 [1.55–4.92] and AOR = 2.13 [1.16–3.89], respectively). Geographic variability in use of modern contraceptives may be a result of the different non-governmental organizations (NGOs) operating in the three study districts within the region of Dosso. A network of international and local NGOs were operating in the region of Dosso during the data collection time period, including Pathfinder International, World Vision, PSI, SongES, Lafia Matassa, Kaidia, and Kebon. These NGOs all recruited *relais* as part of the implementation of their community health programs. We know that different NGOs focused their *relais* training on different health topics; for example, Pathfinder International and PSI focused their *relais* training on family planning while World Vision and Kebon focused their *relais* training on child health. In villages of Doutchi and Loga districts, Pathfinder International, Lafia Matassa, and PSI were all implementing family planning health projects in the community. The strong family planning focus among these NGOs in Doutchi and Loga districts may explain why study participants residing in these districts were positively associated with current use of modern family planning.

This study has several strengths and weaknesses that are important to note. With so few family planning research studies conducted in Niger, there is a knowledge gap and dearth of family planning evidence from the country that ranks at the bottom of the Human Development Index. To our knowledge, no studies have explored patterns between CHW visits and use of contraception among young married women in Niger. This quantitative study conducted among young married women offers new insights and perspectives from a country that has low investments in healthcare, low access and utilization of modern contraceptives, and the world’s highest fertility rate. In addition, the study’s robust quantitative analysis that explores the association between *relais* visits and current use of modern contraceptives through multivariate regression analysis, contributes to the important discussion of CHWs improving access to family planning services in Niger and other LMICs where access to care remains an important public health issue.

Despite the strengths of the study, there are several limitations to highlight. First, this was a cross-sectional study which limits assessment of the temporal relationship between exposure and outcome. Without longitudinal data, it is not possible to establish causal effect of *relais* visits on current use of modern contraceptives. In addition, this was an observational study looking at CHW exposure in rural Niger from May to August 2016. Since *relais* in Niger are trained and supervised by different NGOs—which are often driven by donors to focus on specific health topics—there may be variability in the quality of family planning counseling provided by *relais* which could affect the uptake of modern contraceptives among study participants. Social desirability bias might also be a concern due to the sensitive nature of the questions asked in the survey. Family planning and contraceptive use are sensitive topics in conservative settings; therefore, participants may answer a survey question in a way that they assume will be viewed more favorably by the research team. Lastly, the generalizability of the findings may be limited to only the three rural districts in the region of Dosso where this study was conducted, though could potentially be generalizable to other rural districts that are culturally or geographically similar to the study catchment area.

## Conclusion

Results from this study contribute to the growing knowledge base around CHW and family planning in LMICs, and provide additional evidence for the positive effects of CHWs on current use of modern methods of contraception. Community health workers provide an integral link between communities and the public health system, providing access to essential family planning services in rural and hard-to-reach areas. Study results show that CHW visits were positively associated with current use of modern contraceptive methods among young married women in Niger. The implication of the study findings suggests that trained CHWs in Niger can result in increased access and utilization of modern contraceptives among young married women. Although the majority of young married women found CHW visits helpful, CHW visits to nulliparous women were rare in this study population. Results also show that working young married women were less likely to currently use modern contraceptives. Additional mixed-methods research is needed to develop cost-effective, socially appropriate, and bias-free family planning interventions that allow CHWs to support working adolescents and nulliparous women in Niger.

## Abbreviations

AOR: Adjusted Odds Ratio
AOR: Community Health Worker
AOR: Emergency Contraception
IUD: Intrauterine Device
LAM: Lactational Amenorrhea Method
LMICs: Low- and Middle-Income Countries
SDGs: Sustainable Development Goals
